# The menstrual cycle affects recognition of emotional expressions: an event-related potential study

**DOI:** 10.12688/f1000research.11563.1

**Published:** 2017-06-08

**Authors:** Madoka Yamazaki, Kyoko Tamura

**Affiliations:** 1 Division of Health Science, Daito Bunka University, 560 Iwadono, Higashimatsuyama, Saitama, 355-8501, Japan

**Keywords:** menstrual cycle, late luteal phase, premenstrual syndrome, ERP, N170, emotion

## Abstract

Background: Several studies have investigated the relationship between behavioral changes and the menstrual cycle in female subjects at a reproductive age. The present study investigated the relationship between the menstrual cycle and emotional face recognition by measuring the N170 component of ERPs.

Methods: We measured N170 of twelve women in both follicular phase and late luteal phase who were presented with human facial expressions as stimuli (happy and angry).

Results: In the follicular phase, participants showed a significantly larger response to happy male facial expressions. In the late luteal phase, participants had longer reaction times to all emotional stimuli, and a significantly reduced response to happy faces, especially happy male facial expressions (P<0.001).

Conclusions: Our findings suggest that the menstrual cycle modulates early visual cognitive processing, and highlight the importance of considering the menstrual cycle phase in studies that investigate emotion and cognition.

## Introduction

In cognitive neuroscience, gender differences have been discussed since the late 1990s. This is partly due to the increasing amount of reports showing the gender differences of brain structure ^20, 38, 42, 45, 46^ and metabolism ^1, 36^. For instance, the corpus callosum is larger in women than in men ^2^, and so are the left cortical language-associated regions ^20, 38, 46^. Several behavioral studies have also shown differences between genders, such that women showed higher performance on verbal and memory tasks ^13, 31^, whereas men were excellent at spatial tasks ^12, 17^. Many studies have revealed that the differences in brain anatomy correlated with the behavioural differences between genders, by integrating brain imaging with cognitive tasks ^2, 19, 25^.

Females at reproductive age experience dynamic changes in levels of sex hormones (e.g. estrogen and progesterone) every menstrual cycle. Premenstrual syndrome (PMS) symptoms include mood and behavioral changes such as irritability, depression, mood swings, fatigue and food cravings that develop during the luteal phase within a few days of menstruation. PMS occurs in up to 75% of females that are at a reproductive age ^24, 41^. Although the etiology of PMS is unclear, the hormonal shift from estrogen to progesterone may cause of some of the symptoms of PMS, as this hormonal shift affects the level of serotonin and serotonergic antidepressants that are causative for both the physical and psychological symptoms ^23, 37^.

Several studies have investigated the relationship between behavioral changes and the menstrual cycle in female subjects at a reproductive age. Dreher *et al.* ^14^ reported that women in the follicular phase of their cycles showed higher activation in the orbitofrontal cortex and amygdala than they did during the luteal phase during a gambling task. Slyepchenko *et al.* ^40^ showed that subtle working memory and selective attention impairment occurred more frequently in women with moderate to severe PMS than women in with mild or no PMS symptom. These studies were concordant with symptoms of PMS (difficulty concentrating, lowered performance, lowered judgement). In contrast, Eggert L *et al.* ^16^ reported a kind of paradoxical effect, where women with PMS in the luteal phase of their menstrual cycle showed a greater emotional stroop effect with respect to picture and facial stimuli, compared to a control group. It still remains poorly understood how the menstrual cycle that provokes the changes in sex hormone levels affects emotional cognition.

Event-related potential (ERP) studies have been used for investigating the attention and emotional effects produced with facial stimuli. N170 is an ERP component showing a negative peak at around 140–200ms post-stimulus in the posterior temporal region, and is thought to reflect the detection and global processing of facial images ^7, 10, 15, 22^. It is also thought to be sensitive to the emotion displayed in facial expressions ^4, 8, 36^. Several authors have reported gender differences in N170 that females show greater response to facial stimuli than males ^11, 26, 43^. The N170 is also affected by neurological/psychiatric conditions ^9, 44^. However, no studies have addressed the relationship between N170 and the menstrual cycle, measured with emotional facial expressions.

The present study was conducted to investigate whether the menstrual cycle affects the N170 elicited by the emotional facial expressions. We compared results from the follicular phase and the late luteal phase.

## Methods

### Participants

Twelve female, right-handed students participated in this study with a mean age of 21.6±2.0 (mean ± SD). All participants had normal or corrected to normal vision and regular menstrual cycles between 25 and 33 days with no history of neurological or psychiatric illness.

Each female participant was examined both during the follicular phase (9–12, mean 10.1 days after the first menses) and late luteal phase/premenstrual phase (7-3, mean 4 days before the first day of menses). Half of the participants were examined firstly in their follicular phase to avoid the test–retest effect. All experimental sessions were conducted between the 13:00 and 18:00 to control for the effects of circadian rhythm.

### Consent

This study was approved by Daito Bunka University research ethical committee (K14-008) and written informed consent was obtained from all the participants before the experiment.

### Assessment of menstrual cycle phase

***1. Salivary hormone measurements.*** Salivary estradiol and progesterone (4-pregene-3, 20-dione) were measured using Sal metrics, LLC (State College, PA) ELISA kits and measured optically using xMarkmicroplate spectrophotometer (Bio-Rad, Tokyo, Japan). Approximately 10 minutes after their arrival, participants provided a 1mL saliva sample using the “passive drool” collection method.

***2. Menstrual Distress Questionnaire (MDQ).*** ^33^ A Japanese version of the MDQ translated by the authors was given to participants during both their follicular phase and late luteal phase / premenstrual phase, to evaluate their psychological and physiological status (see Supplementary File S1 for the original questionnaire and Supplementary File S2 for the translated questionnaire in Japanese). The MDQ consists of 47 items which are grouped into eight subcategories: pain, water retention, autonomic reaction, negative affect, impaired concentration, behavioral change, arousal, and control. Participants were required to rate their symptoms using a four-point scale ^1– 4^, ranging from “no experience of symptoms” to “severe” on 47 items.

### Experimental procedures

Participants were seated on an armchair, and a PC screen was placed in front of them at a distance of 80 cm. Participants were asked to respond as fast as possible by pressing the left mouse button with their right index finger when the human facial expression (happy or angry) appeared.

***1. Stimuli.*** Stimuli consisted of pictures of 24 different adult faces (12 male and 12 female), that were obtained from the Karolinska Directed Emotional Faces ^28^. The pictures were shown upright, adjusted to a width of 60 mm and height of 90 mm, and presented on a black background. Three types of facial expression (neutral, happy and angry) were displayed. Faces were displayed for 400 ms, and then a white fixation cross on a black background was displayed lasting randomly between 1300 and a1600 ms. Stimulus delivery was controlled by the presentation software Neurobehavioral systems, version 18.0 (Albany, CA).

Presentation of stimuli occurred in four blocks. In each block, 24 pictures with expressions of emotion (12 happy and 12 angry), and 96 pictures with neutral expression were selected at random, resulting in a total of 480 trials. Error rate and response time were recorded.

***2. ERP recording.*** EEGs were recorded with Ag-AgCl electrodes and electrodes were placed according to the 10–20 system using a Neurofax EEG-1200 (Nihon Kohden, Tokyo, Japan). Electrode impedance was kept < 5kΩ. The amplifier bandpass was 0.1–40 Hz and sampled with a digitization rate of 500Hz.

***3. Data analysis.*** The continuously recorded data were divided into epochs of 900 ms in length, starting 100 ms before stimulus onset. EEGs for the happy and angry facial expressions were averaged separately using the EMSE software suite version 5.52 (Source Signal Imaging, San Diego CA). Tests with wrong responses, or eye blinks, lateral eye movements, or muscle discharges which showed over 100μV were excluded. We analyzed the peak amplitude, latency of interest of ERP components, N170 at posterior temporal head region, T5 and T6 between 140 and 200 ms post-stimulus.

***4. Statistical analysis.*** Statistical tests involved performing paired t-tests using SPSS version 19.0 (SAS Institute Inc., Chicago). A value of p<0.05 was taken to indicate statistical significance.

## Results

### Salivary hormone measurements

The salivary concentrations of 17β-Estradiol and progesterone (4-pregene-3, 20-dione) are presented in Table 1. 17β-Estradiol was higher in the follicular phase and progesterone was significantly higher (t(11)=7.11, *p*<0.05) in the late luteal phase.

**Table 1.** Salivary hormone concentrations.

	follicular phase		late luteal phase	
	mean	S.D. (pg/ml)	mean	S.D. (pg/ml)
17β-Estradiol	2.63	1.13	1.97	1.22
Progesterone	100.65	79.28	262.40	87.27

### Menstrual Distress Questionnaire (MDQ)

Mean MDQ scores for female participants in the follicular phase and late luteal phase are presented in Table 2. Participants in the late luteal phase of their menstrual cycle showed significantly higher scores for pain, concentration, behavioral changes, water retention and negative affect (t(11)=6.41, 4.81, 4,63, 4,66, 3,47, 6,11, all *p*<0.05) compared to when they were in the follicular phase.

**Table 2.** Mean MDQ scores of female participants in follicular phase and late luteal phase.

	Follicular phase		Late luteal phase	
	mean	S.D.	mean	S.D.
Pain	0.75	0.96	4.27	1.49
Concentration	0.75	0.50	5.64	3.85
Behavioral change	1.75	0.96	6.73	3.61
Autonomic reaction	0.00	0.00	0.18	0.60
Water retention	0.00	0.00	3.91	2.02
Negative affect	0.75	0.96	5.91	4.23
Arousal	2.00	1.83	0.36	0.67
Control	0.25	0.50	0.18	0.40
total score	6.25	1.71	27.18	12.58

### Behavioral data

**Figure 1.**
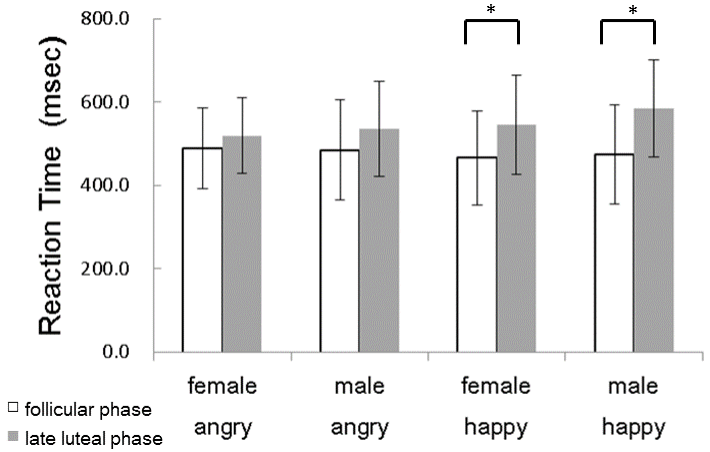
Reaction times to facial expressions of emotion, displayed separately for each emotion. *: *p*<0.01.

The average error rate across all conditions (male/female, happy/angry) was below 1.5% in both the follicular phase and the late luteal phase. Figure 1 shows the mean (±SD) reaction times (RTs) of participants to the target stimuli (happy/angry facial expressions). Participants in the follicular phase of their menstrual cycle responded more quickly to all stimuli than when they were in their late luteal phase. Participants in their late luteal phase showed significantly longer RTs for both male (t(11)=2.99, *p*<0.05) and female (t(11)=2.84, *p*<0.05) happy faces.

### N170

**Figure 2.**
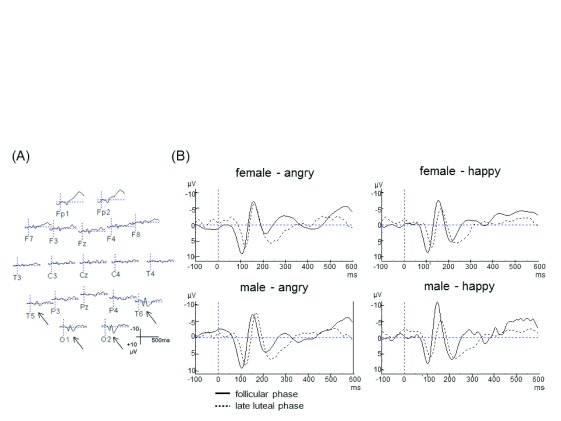
( **A**) ERP grand averages for the emotional facial stimuli. The N170 component localized at the posterior-temporal and occipital electrodes is indicated with an arrow. ( **B**) N170 component grand averages for each of the emotional facial stimuli at the T6 electrode.

**Figure 3.**
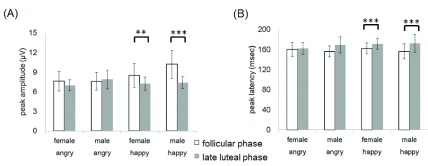
N170 component peak latency ( **A**) and peak amplitude ( **B**). **: *p*<0.01, ***: *p*<0.001.

**Figure 4.**
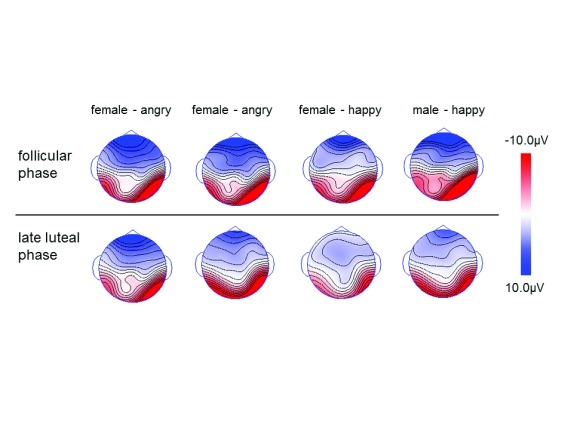
Topographic maps for the N170 component for each of emotional facial stimuli at each peak latency. Upper panel: follicular phase; lower panel: late luteal phase.

Figure 2A shows the ERP grand averages for the facial expressions of emotion (happy and angry) from all participants. N170 was recorded in posterior-temporal and occipital electrodes. Figure 2B shows the ERP grand averages separately for each stimuli at the T6 electrode. Participants in the follicular phase showed higher N170 amplitude (-8.49 *μ*V) than in the late luteal phase (-6.13 *μ*V) for happy female facial expressions (t(11) = 4.31, *p*<0.01) ( Figure 3A). A similar effect was seen for happy male facial expressions (10.9 *μ*V in follicular phase, 6.39 *μ*V in late luteal phase) (t(11) = 7.09, *p*<0.001) ( Figure 3A). The amplitude for both female and male angry facial expressions did not differ between phases of the menstrual cycle ( Figure 3A, Figure 4). Participants in follicular phase showed shorter peak latency of the N170 component irrespective of the type of stimulus, when compared to the late luteal phase. There were significant differences in latency of the N170 component observed between follicular phase and late luteal phase using both happy male facial expressions and happy female facial expressions as stimuli (t(11)= 4.49 / 6.04, *p*<0.001) ( Figure 3B).

Raw data for ERP grand averages, for the target stimuli (angry/happy facial expressions), recorded from all participants The ERP grand average waveforms were re-referenced offline to the average of the left and right mastoids, filtered at 1.0–15 Hz and calculated separately for non-target (neutral face) and target (angry/happy face) stimuli and electrode site, with reference to a 200ms baseline preceding stimulus onset. Click here for additional data file. Copyright: © 2017 Yamazaki M and Tamura K 2017 Data associated with the article are available under the terms of the Creative Commons Zero "No rights reserved" data waiver (CC0 1.0 Public domain dedication).

Raw data for the N170 component grand averages for each of the emotional facial stimuli at the T6 electrode The ERP waveforms were averaged separately for each target stimuli (female/male, angry/happy) in each menstrual phase. T6 electrode activity was extracted as N170 was being recorded. Click here for additional data file. Copyright: © 2017 Yamazaki M and Tamura K 2017 Data associated with the article are available under the terms of the Creative Commons Zero "No rights reserved" data waiver (CC0 1.0 Public domain dedication).

Raw data for the averaged ERP waveforms for each target stimuli (female/male, angry/happy in follicular/late luteal phase), with 19 electrodes and exported to a separate sheet The data was used to create 2-D voltage topographic maps, by calculating the voltage distribution for the N170 component for each of emotional facial stimuli at each peak latency, with EMSE software suite (Source Signal Imaging, San Diego, CA). Spherical spline interpolation was applied. Click here for additional data file. Copyright: © 2017 Yamazaki M and Tamura K 2017 Data associated with the article are available under the terms of the Creative Commons Zero "No rights reserved" data waiver (CC0 1.0 Public domain dedication).

## Discussion

The present study investigated the effect of the menstrual cycle on emotional facial recognition, by measuring ERPs. The most important findings in this study were the effects of the menstrual cycle on behavioral data (RTs) and the N170 component of ERPs.

We expected participants in late luteal phase to respond to the human facial expressions more slowly compared to participants in the follicular phase, as indicated by previous studies ^3, 27, 30^. Significantly longer RTs to happy faces were observed in the late luteal phase than in the follicular phase. Lord T and Taylor K ^27^ reported that women scored lower in concentration tasks in the late luteal phase and Maki PM *et al.* ^30^ also reported the same pattern in performance of motor skill tasks. Our results are consistent with these studies, in which lower performances during late luteal phase were caused by the changes of estrogen level.

The N170 recorded between 140 and 200ms in the lateral temporal region showed faster response in the follicular phase than in the late luteal phase (pre-menstrual phase), irrespective of facial expression. Additionally, the N170 response to happy facial expressions was larger in amplitude in the follicular phase than in the late luteal phase, and also larger in amplitude for male facial stimuli than for female facial stimuli, thus showing for the first time an effect of the menstrual cycle on early components of visual evoked potentials.

Several studies have reported that N170 amplitude can be modified by facial expressions of emotion, especially fearful expressions ^6, 39^. In the present study, the amplitude of the N170 component was significantly larger in response to happy male facial expressions than in response to angry facial expressions in the follicular phase, and slightly larger in response to angry male facial expressions in the late luteal phase. There are several potential explanations for these findings; it has been suggested that stimulus “intensity” may be an important variable in determining N170 amplitude ^50^. Thus, the differences in N170 amplitude elicited by the emotional faces in the present study and other studies may be due to the fact that the emotional faces may be more “intense” or “provocative” to the brain than the other faces.

The present study also found a larger N170 amplitude, especially in response to happy male facial expressions in the follicular phase. This finding may be interpreted as an effect of the existence of an opposite-/same-sex bias in face processing. Several studies have shown that individuals respond more quickly and strongly to attractive faces of the opposite sex than to the same sex ^9, 21, 34^. Therefore, the participants in the follicular phase of their menstrual cycle showed the largest response to man happy face of all stimuli.

Participants in the late luteal phase showed a decreased N170 amplitude and a significantly reduced response to happy facial expressions, compared to the same participants in the follicular phase. As expected, participants in the late luteal phase reported significantly increased scores in MDQ ( Table 2), while the same participants in the follicular phase showed lower scores or absent symptoms. Several researchers have investigated the menstrual effects on cognitive function with ERPs ^3, 29, 47, 49^. They reported that women in the follicular phase showed decreased response (longer latency and smaller amplitude) in the cortical processing of visual stimuli compared to in late luteal phase. The N170 component is also negatively modulated by the psychiatric condition; for instance, high anxiety or a depressive state will influence its properties ^5, 48^.

The decreased response, especially to happy facial expressions, may be the due to the lack of positivity bias, but may also be due to there being a reduced perception of positive stimuli, caused by changes in ovarian hormone levels in the late luteal phase that result in attention deficits.

In summary, this is the first study to provide electrophysiological evidence showing the effects of the menstrual cycle on emotional facial recognition, with the N170 component reflecting early visual processing. Participants in the follicular phase showed a greater response to happy male facial expressions; and participants in late luteal phase (pre-menstrual phase) showed a suppressed response to human facial expressions. These findings highlight the importance of considering the menstrual cycle phase in studies that investigate emotion and cognition.

## Data availability

The data referenced by this article are under copyright with the following copyright statement: Copyright: © 2017 Yamazaki M and Tamura K

Data associated with the article are available under the terms of the Creative Commons Zero "No rights reserved" data waiver (CC0 1.0 Public domain dedication). 

**Dataset 1: Raw data for ERP grand averages, for the target stimuli (angry/happy facial expressions), recorded from all participants.** The ERP grand average waveforms were re-referenced offline to the average of the left and right mastoids, filtered at 1.0–15 Hz and calculated separately for non-target (neutral face) and target (angry/happy face) stimuli and electrode site, with reference to a 200ms baseline preceding stimulus onset.

10.5256/f1000research.11563.d163699 ^51^

**Dataset 2: Raw data for the N170 component grand averages for each of the emotional facial stimuli at the T6 electrode.** The ERP waveforms were averaged separately for each target stimuli (female/male, angry/happy) in each menstrual phase. T6 electrode activity was extracted as N170 was being recorded.

10.5256/f1000research.11563.d163700 ^52^

**Dataset 3: Raw data for the averaged ERP waveforms for each target stimuli (female/male, angry/happy in follicular/late luteal phase), with 19 electrodes and exported to a separate sheet.** The data was used to create 2-D voltage topographic maps, by calculating the voltage distribution for the N170 component for each of emotional facial stimuli at each peak latency, with EMSE software suite (Source Signal Imaging, San Diego, CA). Spherical spline interpolation was applied.

10.5256/f1000research.11563.d163701 ^53^

## Supplementary material

**Supplementary File S1: Original Menstrual Distress Questionnaire (MDQ).**

**Supplementary File S2: Menstrual Distress Questionnaire (MDQ), translated by the authors into Japanese.**
