# Supplementary material for: The menstrual cycle affects recognition of emotional expressions: an event-related potential study
Source: F1000Res. 2017 Jun 8;6:853. [Version 1] doi: 10.12688/f1000research.11563.1 (PMC5558101; doi:10.12688/f1000research.11563.1)
Supplement: Peer review history

# Peer review: Referee response for version 1

**DOI:** 10.5256/f1000research.12492.r23420

**Published:** 2017-08-15

**Authors:** Don M. Tucker

**Affiliations:** 1 Electrical Geodesics, Inc., Eugene, OR, USA

This is a well-organized and well-written study of the important topic of the neural and psuychological effects of menstrual hormonal variation.  The observation of both behavioral (reaction time) and event-related potential measures suggest that the neural changes may be important for everyday behavior and emotional responses.  

 What was the recording reference?  This is important for interpreting the ERP waveforms.  It appears the plots are baseline corrected; is the large ramp over frontal polar channels a result of this?  It looks like there was a large negativity before the stimulus, and that the positive ramping is actually a recovery following a large stimulus-preceeding-negativity that returns to baseline during the perceptual process.

 Addressing these minor points will improve this already solid manuscript.

I have read this submission. I believe that I have an appropriate level of expertise to confirm that it is of an acceptable scientific standard.

# Peer review: Referee response for version 1

**DOI:** 10.5256/f1000research.12492.r24701

**Published:** 2017-08-02

**Authors:** Shunsuke Takagi

**Affiliations:** 1 Department of Psychiatry and Behavioral Sciences, Graduate School of Medical and Dental Sciences, Tokyo Medical and Dental University, Tokyo, Japan

This manuscript reports response and ERP (N170) difference to human facial expression in the different phase of  menstrual cycle of women. The study is well designed and the result is clear and meaningful. However, the authors should address some issues.

 #1 The cause of the result should be clarified or discussed.

 The result (slowed reaction time and enlarged N170 in late luteal phase for emotional stimuli) is impressive and apparently affected by menstrual cycle. However, what factors of the menstrual cycle did affect this data? As the author pointed out, menstrual cycle has many aspects. It is caused by several types of hormones and their cyclic increase and decrease. Such hormones cause emotional changes, fluid balance change etc. during menstrual cycle. Is the result caused by hormonal changes directory or by emotional changes driven by hormones? This matter should be clarified or discussed better.

 #2 Method to obtain N170 should be clarified.

 The method to obtain N170 (recording ERP during presentation of emotional face) is not noted in the method clearly.

I have read this submission. I believe that I have an appropriate level of expertise to confirm that it is of an acceptable scientific standard.
